# A case of adrenal oncocytoma: reviewing the literature of radiological finding

**DOI:** 10.1093/bjrcr/uaae029

**Published:** 2024-08-30

**Authors:** Maho Sakano, Yukari Wakabayashi, Natsuhiko Shirota, Yoshio Ohno, Aoi Suketa, Toshitaka Nagao, Kazuhiro Saito

**Affiliations:** Department of Radiology, Tokyo Medical University, Shinjuku-ku, Tokyo, 1600023, Japan; Department of Radiology, Tokyo Medical University, Shinjuku-ku, Tokyo, 1600023, Japan; Department of Radiology, Tokyo Medical University, Shinjuku-ku, Tokyo, 1600023, Japan; Departmet of Urology, Tokyo Medical University, Shinjuku-ku, Tokyo, 1600023, Japan; Department of Diagnostic Pathology, Tokyo Medical University, Shinjuku-ku, Tokyo, 1600023, Japan; Department of Diagnostic Pathology, Tokyo Medical University, Shinjuku-ku, Tokyo, 1600023, Japan; Department of Radiology, Tokyo Medical University, Shinjuku-ku, Tokyo, 1600023, Japan

**Keywords:** adrenal adenoma, adrenal oncocytoma, oncocytoma, MRI, CT

## Abstract

Oncocytoma is a tumour that predominantly occurs in the kidneys and salivary glands. Only approximately 200 cases have been reported to be of adrenal origin to date, and only a few reports about its radiological findings have been published so far. Herein, we present the CT and MRI findings of an adrenal oncocytoma observed in a patient suspected of having mitochondrial abnormalities, along with the pathological findings. The tumour was roughly classified into three areas: a hypercellular region, a region containing fibrous tissue, and an oedematous region. These corresponded to the restricted diffusion area on the apparent diffusion coefficient map, the gradually enhanced area at the secretory phase on contrast-enhanced CT scan, and the obvious hyperintensity on the T2-weighted image, respectively. We also discuss these findings in the context of previously reported radiological findings in the literature. Diagnosing adrenal oncocytoma through imaging is challenging, and it is crucial to consider the possibility of malignancy while making the differential diagnosis. Small-sized homogenous tumours may be hard to differentiate from lipid-poor adenomas, while larger inhomogeneous ones are hard to distinguish from adrenal cancer.

## Clinical presentation

A 45-year-old Japanese female was programmed for cochlear implant surgery due to bilateral hearing impairment during the Coronavirus disease 2019 (COVID-19) pandemic. All the patients who required surgery were assessed for COVID-19 during the pandemic at our hospital. Numerous patients presented with subclinical COVID-19 infection. Hence, CT scan was conducted to facilitate a safe intubation anaesthesia to prevent spread of the infection. A CT scan was performed, and a left upper abdominal tumour was found incidentally. The patient’s medical history included bilateral hearing impairment, pulmonary artery stenosis, type 1 diabetes mellitus, and short stature (height: 142 cm). As her mother also had bilateral hearing impairment and type 1 diabetes mellitus, she was suspected of having mitochondrial abnormalities without a definitive diagnosis. The patient did not present with symptoms related to the adrenal mass. Blood examination revealed mild elevations of blood glucose and HbA1c levels. No other abnormality was noted except for an increase in Dehydroepiandrosterone sulfate (DHEA-S). Abdominal CT and MRI were performed for further exploration.

## Imaging findings

**Figure 1.**
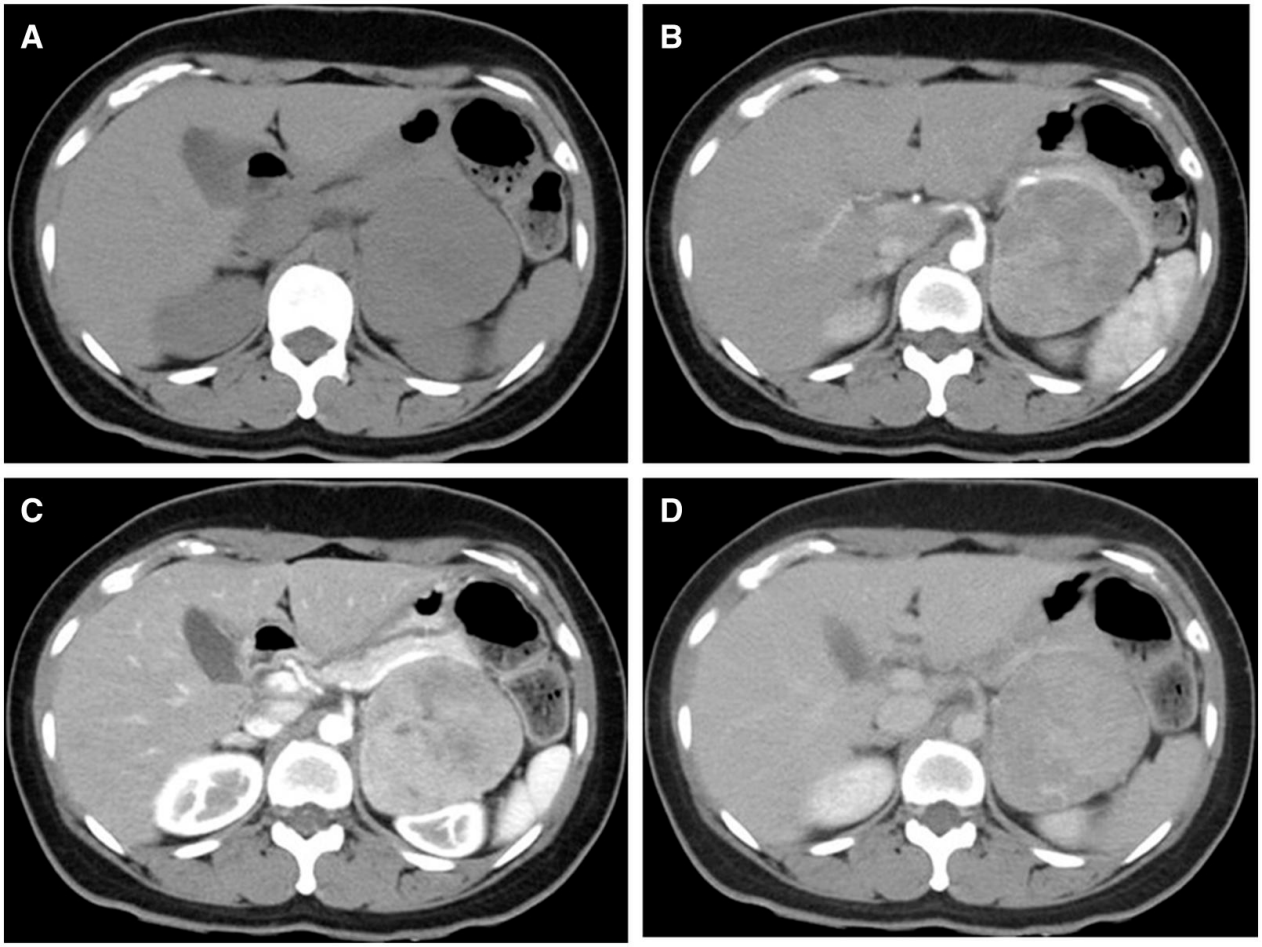
(A) Pre-contrast, (B) early arterial, (C) late arterial, and (D) equilibrium-phase CT. On pre-contrast CT, the tumour is isodense compared to the muscle, and there is no calcification. The dorsal portion of the tumour has a peak of contrast in the late arterial phase, with washout in the equilibrium phase. The ventral portion of the tumour gradually enhanced until the equilibrium phase.

A pre-contrast-enhanced abdominal CT (Figure 1A) revealed a left adrenal tumour of 7 cm diameter with well-defined margins. Most parts of the lesion showed isodensity compared with skeletal muscles; slight hypodensity was observed in the central region, and no calcification was observed. A left renal tumour was suspected initially. Thus, contrast-enhanced CT scan was performed using the renal lesion protocol (early arterial, late arterial, and 5-min secretory phases). An inhomogeneous enhancement was observed at the early and late arterial phases (Figure 1B and C, respectively). The ventral and dorsal parts of the lesion had vague differences. The dorsal area presented with peak enhancement at the late arterial phase (Figure 1C), which washed out thereafter (Figure 1D). The ventral area was gradually enhanced at the secretory phase (Figure 1B–D). When the region of interest was set over the whole lesion, the percentage of absolute enhancement washout at the secretory phase rather than at the 15-min delayed phase was 55%. As the CT scan result was inconclusive, MRI was performed.

**Figure 2.**
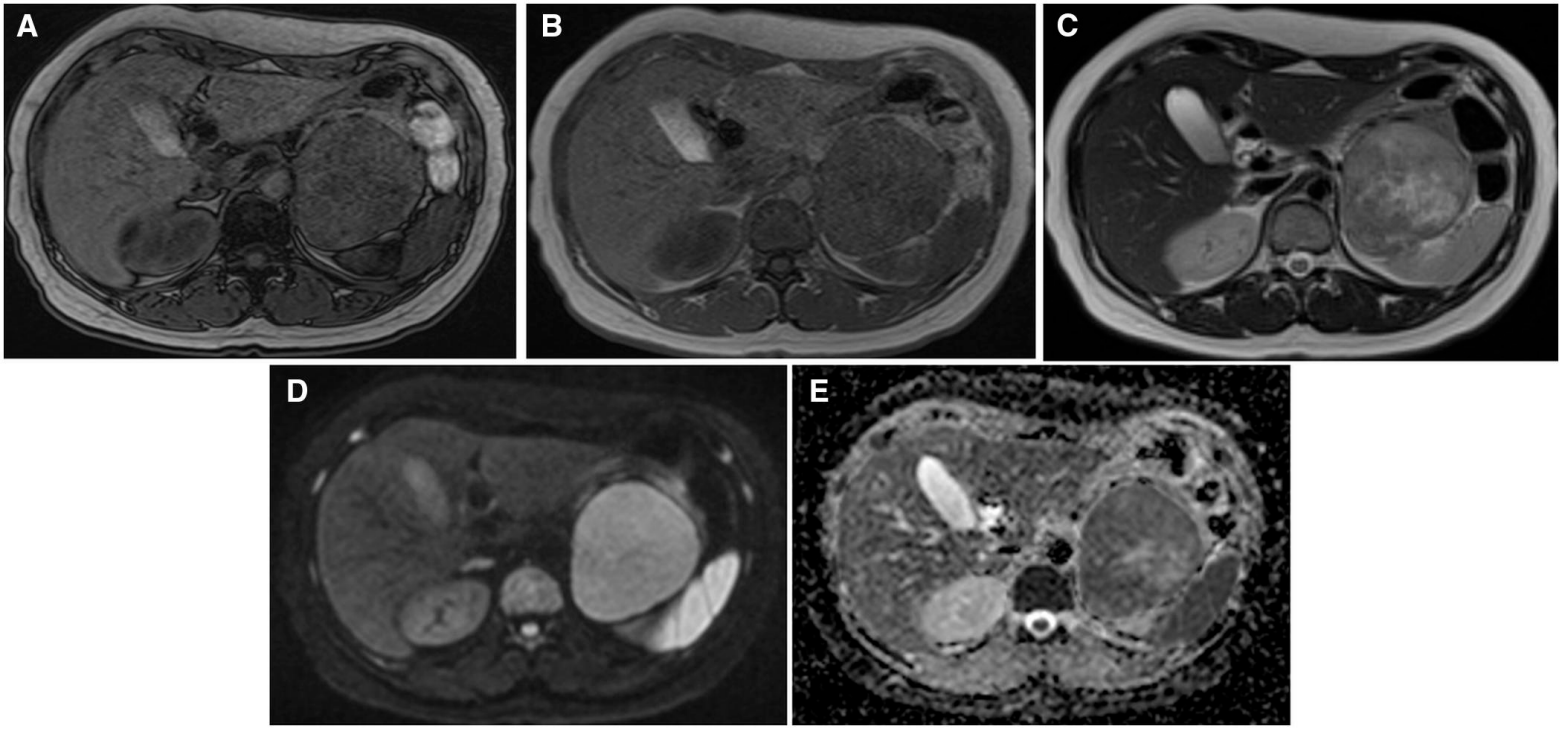
MRI of the tumour. (A) out-of-phase, (B) in-phase, (C) T2WI, (D) DWI *b* = 800, and (E) ADC images. The lesion is almost isointense to muscle on T1WI, without the fat component. It has a slightly higher intensity than muscle on T2WI; however, the dorsal portion of the tumour is slightly lower in signal than the ventral one. The central portion has the highest T2 intensity. The ADC of the dorsal portion is lower than that of the ventral portion. Abbreviations: ADC = apparent diffusion coefficient; T1WI = T1-weighted imaging; T2WI = T2-weighted image.

The lesion was almost isointense on T1-weighted MRI (Figure 2A,B), and the lesion was hyperintensity on T2-weighted imaging compared to muscle tissue (Figure 2C). A part of the lesion was evidently hyperintense on T2-weighted imaging. T2-weighted imaging showed lower signal intensity on the dorsal area than the ventral area, and the area between the ventral and dorsal areas showed obvious hyperintensity. Diffusion-weighted imaging (DWI) showed restricted diffusion (Figure 2D). On the apparent diffusion coefficient map (ADC map), the dorsal area showed lower ADC than the ventral area, indicating more restricted diffusion on the dorsal area of the lesion (Figure 2E). The capsular structure was seen on T2-weighted imaging, and no fatty tissue was detected on chemical shift imaging.

## Treatment and outcome

Based on the radiological findings such as a large lesion size and an inhomogeneous internal density, adrenal cancer could not be ruled out, and surgery was performed.

**Figure 3.**
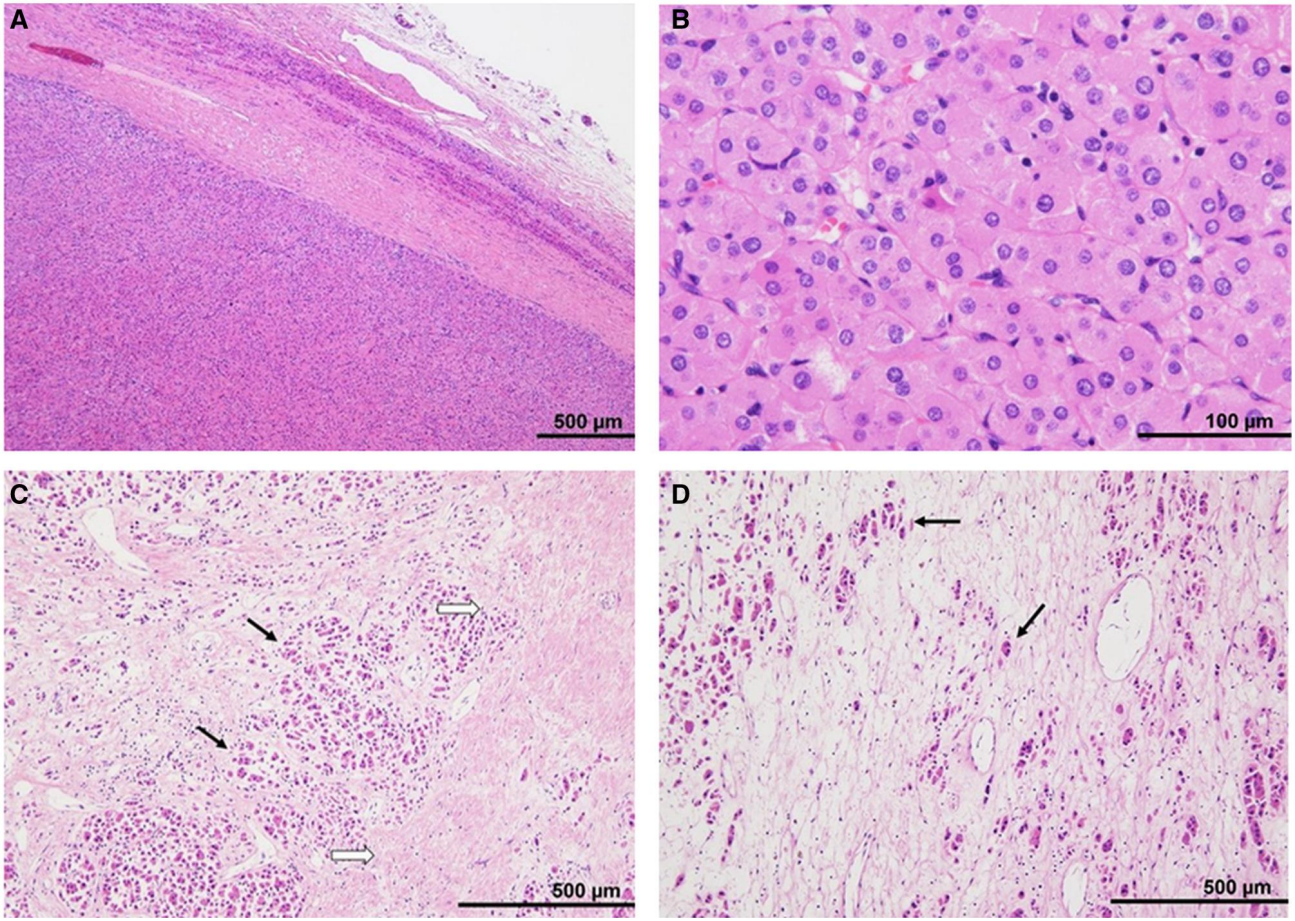
Pathological findings: H-E stain (A) peripheral portion of the tumour, (B) high-power field, (C) ventral portion of the tumour, and (D) dorsal portion of the tumour. (A) The tumour was a well-circumscribed mass encapsulated by a thick fibrous capsule. Compressed non-neoplastic adrenal tissue was seen around the tumour. (B) The tumour cells had eosinophilic cytoplasms and relatively uniform, small round nuclei. (C) The gradually enhanced areas on the equilibrium phase of the enhanced CT revealed fibrosis (black arrows) surrounding the tumour nests (white arrows). (D) In the area with a high signal intensity on T2WI, eosinophilic tumour cells (arrows) were scattered within the oedematous tissues with a low cellular density. Abbreviations: H-E = haematoxylin-eosin; T2WI = T2-weighted image.

The excised lesion, a yellowish solid mass with a smooth surface (macroscopically), measured 7.5 × 6.5 × 6.0 cm. Haematoxylin–eosin staining revealed that the tumour cells with eosinophilic cytoplasms arranged in nests and cord-like patterns and proliferated uniformly. The fibrous capsule was observed peripherally. The nuclei of the tumour cells were relatively uniform and round, and there was no significant cellular atypia and no mitotic figure. The tumour was roughly classified into the following three areas: a hypercellular region, a containing fibrous tissue region, and an oedematous region, which corresponded to the restricted diffusion area on the ADC map, the gradually enhanced area at the secretory phase on contrast-enhanced CT scan, and the obvious hyperintensity on the T2-weighted image, respectively (Figure 3). On immunohistochemical staining, cytokeratin, Melan-A, and Inhibin-α showed partial positivity. Synaptophysin, HMB45, and S100 were negative. The Ki67 positivity rate was approximately 1%. Based on these immunohistochemical staining findings, we diagnosed the patient with adrenal oncocytoma. The postoperative course was good, and the patient neither developed recurrence nor metastasis for 2 years.

## Discussion

Adrenal oncocytoma is considered to be about 20% malignant and 10%-20% functional. It occurs across a wide range of ages, from 15 to 77 years, with a 2.5-fold higher prevalence in females. It is also 3.5 times more common in the left adrenal gland. Its risk factors are not well-known.^1^ Differentiating between benign and malignant adrenal oncocytoma is pathologically and radiologically challenging. Pathological criteria for distinguishing between benign and malignant tumours include the Lin-Weiss-Bisceglia criteria, according to which the presence of at least one major criterion (nuclear pleomorphism, mitotic figures >5/50 high-power fields, venous invasion) indicates malignancy. In comparison, the presence of at least one minor criterion (tumour size >10 cm or weight >200 g, coagulative necrosis, capsular invasion, sinusoidal invasion) suggests borderline malignancy. It is considered benign if none of these criteria are met.^2^ Based on these criteria, the tumour in the present case was considered pathologically benign.

In previous reports, the radiological diagnosis of adrenal oncocytoma has been challenging, especially when it comes to distinguishing it from adrenal cancer. Radiological features that raise suspicion of malignancy include a diameter of at least 5 cm, indistinct margins, irregular shape, and inhomogeneous contrast enhancement.^3^ Therefore, in this case of a 7-cm tumour with slightly heterogeneous internal characteristics, the initial differential diagnosis would typically include adrenal cancer. Reports have revealed that oncocytomas present as an inhomogeneous enhancement on CT scan.^4^^,^^5^ However, only a few studies have mentioned vascularity in these lesions.^5^ The CT scan findings in this case are consistent with those in previously reported cases. As inhomogeneous enhancement at the early and late arterial phases was observed in this case, the lesion might present with a hypervascular region. Nevertheless, these findings are similar to those of adrenal cancer. We hypothesized that it is challenging to obtain a differential diagnosis between oncocytoma and adrenal cancer based on the enhancement pattern at the dynamic phase. Therefore, enhancement washout can be a useful tool. However, only one case of oncocytoma was encountered.

The reports of Khan et al^6^ showed high sensitivity for the percentage enhancement washout, suggesting benignity. They reported that the probability of benignity is higher when the washout percentage is at least 60%. High washout suggests uniformity in vascularity and the extracellular space. In contrast, malignant tumours tend to retain contrast agents in the extracellular space due to increased capillary permeability and cell membrane disruption. Therefore, oncocytomas share a similar vascular behaviour with lipid-poor adenomas, making the differentiation challenging, especially for smaller lesions.^7^^,^^8^ Furthermore, as mentioned above, adrenal oncocytoma sometimes appears as an inhomogeneous lesion. In particular, fibrosis lowers the washout percentage, which makes the tumour similar to a malignant one per that indicator. In present case, the calculated washout percentage was 55%, which is similar to 60%. However, in this case, the percentage washout ratio did not indicate the original percentage washout ratio, and the original method’s threshold could not be accepted as such. Kamiyama et al^9^ proposed an alternative method, which completed at 5 min and 35 s after the administration of contrast media. Subsequently, the absolute washout ratio was used to distinguish lipid-rich adenoma, lipid-poor adenoma, and non-adenoma, which presented satisfactory results. As the protocol of Kamiyama et al^9^ was similar to present study, their method was used for to calculate percentage enhancement washout ratio and the relative percentage washout ratio of the tumour, which were 18% and 11%, respectively. These results significantly indicated malignancy.

A comprehensive case series on the MRI findings of adrenocortical oncocytoma could not be found. Among the previous descriptions related to MRI, the tumours presented with heterogeneous signal intensity in some cases. This heterogeneity reflected haemorrhage and necrosis within the tumour.^4^^,^^5^^,^^10^ However, haemorrhage and necrosis were not observed in the present case. The heterogeneous intensity of the lesion on MRI corresponded to a mixture of fibrosis and oedema pathologically. Renal origin of the tumour was the most frequent radiological finding of oncocytoma. Presence of central scar and radial vessels are characteristics of renal oncocytoma. However, these findings were not observed in the adrenal glands.^1^ Studies on DWI of adrenal oncocytomas are extremely rare. In the present case, a large part of the lesion had high signal intensity on DWI and low signal intensity on the ADC map. These results indicated restricted diffusion and corresponded to the high cellularity of oncocytoma. The pathological findings were in accordance with the DWI findings.

This case presents a rare example of adrenal oncocytoma, and it was discussed primarily based on radiological findings, along with a review of past reported cases. Diagnosing adrenal oncocytoma through imaging is challenging, and it is crucial to consider the possibility of malignancy while making the differential diagnosis. Small-sized homogenous tumours may be hard to differentiate from lipid-poor adenomas, while larger inhomogeneous ones are hard to distinguish from adrenal cancer.

## Learning points

- The radiological findings of adrenal oncocytoma included inhomogeneous enhancement on contrast-enhanced CT scan or MRI and restricted diffusion on diffusion-weighted imaging based on cellularity, fibrosis degree, and oedematous change.
- The radiological findings of adrenal oncocytoma mimic those of adrenal cancer. Hence, these two conditions are challenging to distinguish from each other.

## Informed consent statement

Written informed consent was obtained from the patient for publication of this case report and accompanying images.
